# Complete mitogenome of the entomopathogenic fungus *Tolypocladium cylindrosporum*

**DOI:** 10.1080/23802359.2020.1714495

**Published:** 2020-01-16

**Authors:** Shu Zhang, Yong-Jie Zhang

**Affiliations:** School of Life Science, Shanxi University, Taiyuan, China

**Keywords:** *Tolypocladium cylindrosporum*, mitogenome, Ophiocordycipitaceae

## Abstract

In this study, the complete mitogenome of an entomopathogenic fungus *Tolypocladium cylindrosporum* is assembled and annotated. This circular mitogenome is 34,698 bp in length and consists of two rRNA genes (*rnl* and *rns*), 26 tRNA genes, 14 standard protein-coding genes of the oxidative phosphorylation system, and four intergenic free-standing ORFs. A total of six introns (all group I) were identified in *atp9*, *cob*, *cox1*, and *rnl*, and they may encode ribosomal protein S3, LAGLIDADG or GIY-YIG endonucleases. Phylogenetic analysis based on concatenated mitochondrial protein sequences confirms *T. cylindrosporum* in Ophiocordycipitaceae.

*Tolypocladium cylindrosporum* W. Gams 1971 is a fungus that has been isolated from soil, from asymptomatic plants as an endophyte, and has been shown to be pathogenic to several species of arthropods (especially mosquito larvae) (Scholte et al. 2004; Herrero et al. 2011). It has the potential to be used as a biocontrol agent against mosquito and blackfly larvae (Ekbom 1993; Nadeau and Boisvert 1994). Several biologically active compounds (e.g. fumonisin, tolypin, efrapeptins) were identified from this fungus (Weiser and Matha 1988; Bandani 2005; Zabalgogeazcoa et al. 2018). Its molecular studies, however, are largely limited. As of December 2019, there are only 74 records under *T. cylindrosporum* in the public nucleotide database, and more than 4/5 of them are nuclear ribosomal DNA fragments. Mitochondrial genome (mitogenome) information is not yet available for this fungus. Herein, we present the complete mitogenome of *T. cylindrosporum* ARSEF 963, which was isolated from soil in a spruce-fir forest (elev. 5500-6000 ft) in north Luscar, Alberta, Canada (N53°04′, W117°23′). The strain is obtained from USDA-ARS Collection of Entomopathogenic Fungal Cultures (ARSEF).

Total DNA of ARSEF 963 was randomly sheared to fragments of ∼400 bp, followed by sequencing on an Illumina HiSeq 4000 platform in 2 × 150 bp reads. Mitogenome was *de novo* assembled from clean reads using MITObim (Hahn et al. 2013) and then annotated as described previously (Zhang et al. 2017). Introns in *rnl* and protein-coding genes are named according to proposals suggested by Johansen and Haugen (2001) and Zhang and Zhang (2019), respectively.

The mitogenome of *T. cylindrosporum* (GenBank accession: MN842262) is a circular molecule of 34,698 bp with an AT content of 73.0%. It is rather compact with genic regions (29,672 bp) accounting for 85.5% of the total bases. This mitogenome encodes two ribosomal RNAs (*rnl* and *rns*), 26 tRNAs, 14 conserved proteins of the oxidative phosphorylation system (*nad1*-*6*, *4 L*; *cob*; *cox1*-*3*, and *atp6*, *8*, *9*), and four free-standing ORFs at intergenic regionsf. These tRNA genes code for all 20 standard amino acids. Among them, there are three tRNA genes for methionine with the same anticodon, two tRNA genes for arginine, glycine, leucine, and serine with different anticodons. The majority of tRNA genes are clustered upstream (*trnV*, *I*, *S2*, *W*, *P*) and downstream (*trnT*, *E*, *M1*, *M2*, *L1*, *A*, *F*, *K*, *L2*, *Q*, *H*, *M3*) of the *rnl* gene, and downstream (*trnY*, *D*, *S1*, *N*) of the *rns* gene. For the two commonly-found neighboring gene pairs, *nad3* follows immediately *nad2*, whereas *nad5* overlaps one nucleotide with its upstream gene *nad4L*. The four intergenic free-standing ORFs encode either GIY-YIG endonucleases (*orf221* and *orf296*) or hypothetical proteins (*orf194* and *orf142*). It should be noted that *nad6* overlaps 50 bp at the 3’ end with *trnV*. Sequences at *rns*/*trnY* intergenic region showed weak similarity (Identity 76%; *E* value 2E-30) to *orf221*, but not a complete ORF larger than 300 bp could be identified. This region possibly contains a degenerating GIY-YIG endonuclease.

A total of six introns were identified, including one, one, two, and two in *atp9*, *cob*, *cox1*, and *rnl*, respectively. They are all group I introns but belong to several specific types, namely IA (mL2450 and atp9P181), IB (cox1P212 and cox1P731), IC1 (mL965), and ID (cobP393). Intronic ORFs encode ribosomal protein S3 (in mL2450), LAGLIDADG endonuclease (in cox1P731), or GIY-YIG endonucleases (in remaining introns). Intronic regions (including intronic ORFs) have a total length of 7480 bp.

Phylogenetic analysis based on mitochondrial protein sequences confirms *T. cylindrosporum* as a member of Ophiocordycipitaceae (Figure 1). The fungus is closely related to *Tolypocladium inflatum* and *Tolypocladium ophioglossoides*, and their clustering receives 100% support.

**Figure 1.**
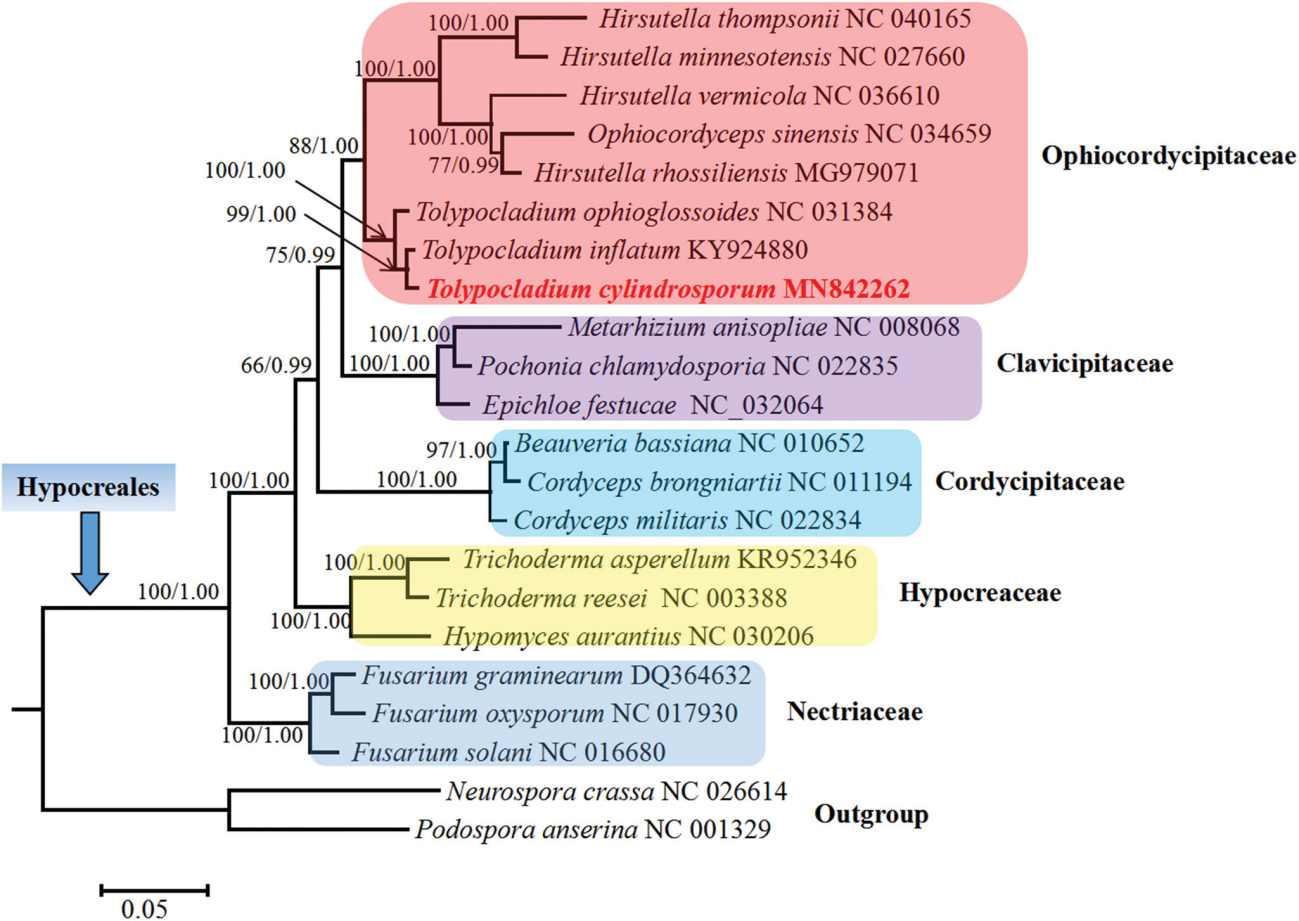
Phylogenetic analysis of Hypocreales species based on 13 concatenated mitochondrial proteins. Concatenated amino acid sequences of Atp8, Atp9, Cob, Cox1, Cox2, Cox3, Nad1, Nad2, Nad3, Nad4, Nad4L, Nad5, and Nad6, a total of 3582 characters, were used. The protein Atp6 was excluded due to its significant phylogenetic conflict with other proteins such as Cob, Cox1, Nad1, Nad4, and Nad5. The tree shown here was the single best topology recovered from the maximum-likelihood (ML) approach as implemented in RAxML v8.2.12, and the topology was quite identical to that recovered from Bayesian inference (BI) as implemented in MrBayes v3.2.7. Support values from ML (*before forward slash*) and BI (*after forward slash*) analyses were given for each node.
